# Health care costs and lost productivity costs related to excess weight in Belgium

**DOI:** 10.1186/s12889-022-14105-9

**Published:** 2022-09-06

**Authors:** Vanessa Gorasso, Isabelle Moyersoen, Johan Van der Heyden, Karin De Ridder, Stefanie Vandevijvere, Stijn Vansteelandt, Delphine De Smedt, Brecht Devleesschauwer

**Affiliations:** 1 Department of Epidemiology and Public Health, Sciensano, Rue J Wytsman 14, 1050 Brussels, Belgium; 2 Department of Public Health and Primary Care, Ghent University, Ghent, Belgium; 3 Department of Applied Mathematics and Statistics, Ghent University, Ghent, Belgium; 4 Department of Medical Statistics, London School of Hygiene and Tropical Medicine, London, United Kingdom; 5 Department of Veterinary Public Health and Food Safety, Faculty of Veterinary Medicine, Ghent University, Merelbeke, Belgium

**Keywords:** Excess weight, Overweight, Obesity, Healthcare costs, Absenteeism costs

## Abstract

**Background:**

This study aimed to estimate annual health care and lost productivity costs associated with excess weight among the adult population in Belgium, using national health data.

**Methods:**

Health care costs and costs of absenteeism were estimated using data from the Belgian national health interview survey (BHIS) 2013 linked with individual health insurance data (2013–2017). Average yearly health care costs and costs of absenteeism were assessed by body mass index (BMI) categories – i.e., underweight (BMI < 18.5 kg/m^2^), normal weight (18.5 ≤ BMI < 25 kg/m^2^), overweight (25 ≤ BMI < 30 kg/m^2^) and obesity (BMI ≥ 30 kg/m^2^). Health care costs were also analysed by type of cost (i.e. ambulatory, hospital, reimbursed medication). The cost attributable to excess weight and the contribution of various other chronic conditions to the incremental cost of excess weight were estimated using the method of recycled prediction (a.k.a. standardisation).

**Results:**

According to BHIS 2013, 34.7% and 13.9% of the Belgian adult population were respectively affected by overweight or obesity. They were mostly concentrated in the age-group 35–65 years and had significantly more chronic conditions compared to the normal weight population. Average total healthcare expenses for people with overweight and obesity were significantly higher than those observed in the normal weight population.

The adjusted incremental annual health care cost of excess weight in Belgium was estimated at €3,329,206,657 (€651 [95% CI: €144-€1,084] and €1,015 [95% CI: €343–€1,697] per capita for individuals with overweight and obesity respectively). The comorbidities identified to be the main drivers for these incremental health care costs were hypertension, high cholesterol, serious gloom and depression. Mean annual incremental cost of absenteeism for overweight accounted for €242 per capita but was not statistically significant, people with obesity showed a significantly higher cost (*p* < 0.001) compared to the normal weight population: €2,015 [95% CI: €179–€4,336] per capita. The annual total incremental costs due to absenteeism of the population affected by overweight and obesity was estimated at €1,209,552,137. Arthritis, including rheumatoid arthritis and osteoarthritis, was the most important driver of the incremental cost of absenteeism in individuals with overweight and obesity, followed by hypertension and low back pain.

**Conclusions:**

The mean annual incremental cost of excess weight in Belgium is of concern and stresses the need for policy actions aiming to reduce excess body weight. This study can be used as a baseline to evaluate the potential savings and health benefits of obesity prevention interventions.

**Supplementary Information:**

The online version contains supplementary material available at 10.1186/s12889-022-14105-9.

## Background

The sustained global increase of overweight and obesity over the last 40 years puts a heavy burden on the health system worldwide [1]. In 2019, excess weight was one of the top three risk factors in terms of attributable death and disability-adjusted life years (DALYs) and was increasing in exposure by more than 1% per year globally [2]. Cardiovascular disease, type 2 diabetes, kidney diseases and neoplasms account for about 90% of the excess weight-related DALYs globally [2]. Excess weight is strongly associated with the occurrence of chronic diseases, impaired health-related quality of life, increased health care and medication spending [3] [4] and a decreased workforce productivity [5].

Despite the disturbing figures in the global obesity prevalence and the related costs, no country or subpopulation was able yet to reverse the upward trend of obesity [1, 6]. Addressing the obesity pandemic requires a multi-sectoral approach across multiple areas of governance and well-defined programs on control and prevention. Along with epidemiological studies, cost of illness studies help to demonstrate the harmful effects of diseases in financial terms. This information, with cost-effectiveness studies, can then be used by policy makers to prioritise the allocation of resources to prevention, treatment and research [7].

In Belgium, as in many high-income countries, average body mass index (BMI) has increased over the past decades among both children and adults. According to the Belgian health examination survey (BHES), in 2018, more than half of the adult population was affected by overweight and 16% was affected by obesity [8]. The latest available cost data reported that in 2010, around €600 million was spent on medical care of obesity related pathologies [9]. In addition, based on a Markov decision-analytic model, a one unit BMI-reduction in the Belgian population affected by overweight and obesity was estimated to result in a societal cost (direct and indirect) saving of €2.8 billion [10].

Considering the importance of this risk factor and the need for updated evidence, this study investigates the burden of excess weight including overweight and obesity among the adult population on annual health care costs and lost productivity costs in Belgium, and investigates to what extent differences in expenditures by BMI differ by socio-demographic characteristics and comorbidity burden.

## Methods

### Data

Individual participant health care costs related to obesity and overweight were obtained by linking two national databases, i.e. the Belgian Health Interview Survey (BHIS) 2013 and the national health insurance data compiled by the Intermutualistic Agency (IMA) 2013–2017. Linkage was performed by means of a National Registry Number. The BHIS was conducted between January and December 2013 among a representative sample of the Belgian population (*N* = 10,828) and comprises data on health status and related health behaviour and determinants. Respondents were recruited following a multistage sampling design, as described in detail elsewhere [11]. Interviews were performed using a face-to-face paper and pencil interviewing, supplemented with a self-administered questionnaire covering more sensitive topics [11]. Health insurance is compulsory in Belgium covering more than 99% of the population. The linked IMA database used for this study comprises aggregated reimbursed health care costs from 2013–2017 for all HIS participants including expenditures for 1) ambulatory care (pharmaceuticals excluded), 2) hospital care, and 3) reimbursed medicines purchased through public pharmacies. The linked IMA database readily included only information on hospital care variable costs (i.e. costs depending on the type of interventions performed during the hospital stay). However, in Belgium, the national health insurance also pays a fixed amount to the hospitals per admitted patient, depending on the type of hospital and treatment. Precise information on these costs was not directly available in the dataset. In order to estimate the fixed part of the total hospital care cost, the hospitalizations per patient per year were multiplied with the average annual 100% per diem cost publicly available by type of hospitalization (per diem costs available through: https://www.riziv.fgov.be/nl/themas/kost-terugbetaling/door-ziekenfonds/verzorging-ziekenhuizen/Paginas/verpleegdagprijzen-ziekenhuizen.aspx). Finally, we summed up the estimated fixed costs with the available variable hospital costs resulting in the total hospitalization costs used in this analysis.

**Fig. 1:**
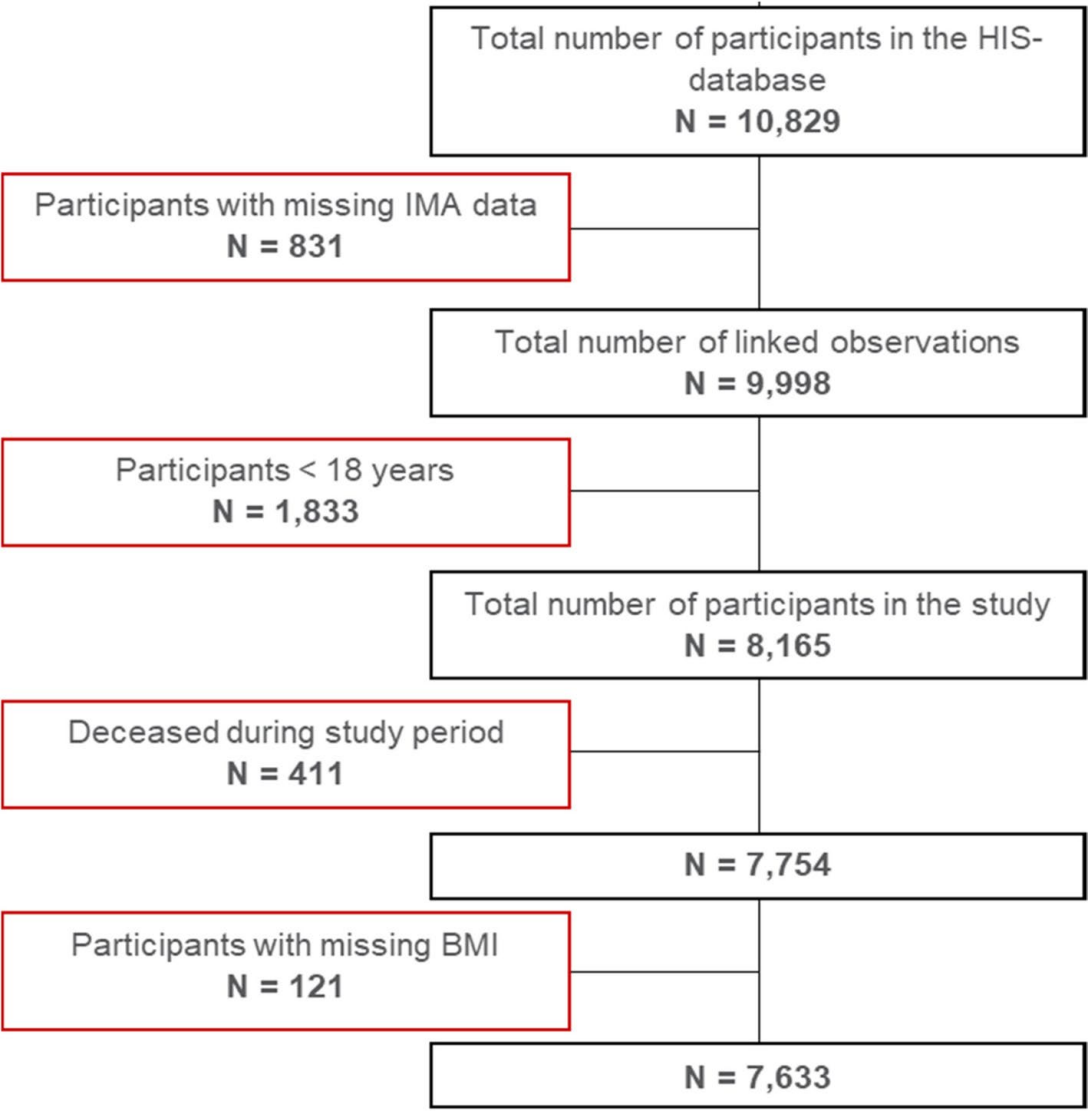
Flow diagram of number of respondents included in the study

The study included the adult population (age ≥ 18 years) who reported weight and height and for whom linkage with health insurance data was possible and were continuously insured from 2013–2017 (latest linkage available). People who deceased during the study period (from their participation to the BHIS until 31/12/2017) were excluded. The final study sample comprised 7,633 participants (Fig. 1).

Health care costs were analysed by BMI category calculated from self-reported weight and height obtained from the BHIS using the classification recommended by the World Health Organization, i.e., underweight (BMI < 18.5 kg/m^2^), normal weight (18.5 ≤ BMI ≤ 24.99 kg/m^2^), overweight (25 ≤ BMI < 30 kg/m^2^) and obesity (BMI ≥ 30 kg/m^2^) [12]. Socio-demographic variables taken into account included age, gender, household educational level (i.e. the highest educational level within the household), and income level (based on the calculated quintiles of the household income), as well as behavioural risk factors with respect to alcohol misuse, smoking, poor dietary quality and physical inactivity. Analyses also concerned information from the BHIS database on the prevalence of 23 major chronic conditions, i.e., asthma, chronic bronchitis, myocardial infarction, coronary heart disease, other serious heart disease, hypertension, high blood cholesterol, stroke, narrowing of blood vessels, arthritis (including rheumatoid arthritis and osteoarthritis), low back pain, neck pain, diabetes, allergy, stomach ulcer, cirrhosis of the liver, cancer, severe headache, urinary incontinence, serious gloom or depression, thyroid problems, and eye disease.

Absenteeism was reported in the BHIS as days absent from work during the 12 months prior to the BHIS interview queried by the following question: “Have you been absent from work during the past 12 months due to health problems? In doing so, take into account any conditions, injuries or other health problems you may have had and which resulted in an absence from work”. Followed by the question: “How many days in total have you been absent from work for the past 12 months due to health problems? If you are unable to indicate this number of days correctly, please give an estimate.”. The question was asked to working individuals only (*N* = 3,857) – individuals that stated to have a paid job at the moment of the interview.

### Analysis

Analyses were conducted in R 4.0.5 [13] taking the design of the survey into account. The sampling design included stratification at the level of the provinces and clustering at the household level, as described in Demarest et al. [11]. Analysis of socio-demographic characteristics and healthcare costs per BMI category were performed using nominal logistic regression for comparison of proportions between BMI categories with normal weight as reference group. Confidence intervals (CI) were computed via the delta method, using the standard errors resulting from the survey analysis.

### Health care costs

Overall health care costs and health care costs by type and by payment modality were calculated per BMI category. Univariate and multivariable regressions with negative binomial distribution and log link were used to explore the extent to which average yearly health care cost was associated with BMI category, socio-demographic characteristics and behavioural risk factors. The univariate model of health care cost in function of BMI-class allowed to estimate the unadjusted incremental health care costs and to evaluate statistical differences in average costs between individuals with underweight, overweight and obesity compared to normal weight individuals.

A “double-selection” approach was used for the selection of the variables to be included in the final multivariable model, based on backward elimination to identify significant variables at the 10% level [14]. The variables were identified in two steps, finding those that predict the dependent variable (costs) and those that predict the independent variables (BMI-class). The final linear regression included the covariates identified in either of the two steps. Candidate explanatory variables included age groups, gender, household educational level, household level of income and some behavioural risk factors such as smoking, alcohol misuse, unhealthy eating behaviour and physical inactivity. This use of double-selection is more likely to detect common causes of BMI-category and costs, and thereby results in more accurate inferences that also acknowledge the uncertainty in the selected variables.

### Indirect costs—Cost of absenteeism

Cost of absenteeism was computed by multiplying the number of days absent from work by the national average labour cost per day. Using the costing year 2010 from Eurostat, the average Belgian labour cost per working day was estimated at €257 (monthly labour cost and assuming 18.8 working days per month (i.e., 52 weeks * 5 working days minus 24 days (legal holidays and agreed extra holidays) minus 10 public holidays) [15]. However, since respondents might have included weekend days in their answer, the total days absent from work was subtracted from the maximum number of working days per year, i.e., 226 days. If this difference was equal to or greater than zero, the answer was kept, else the maximum number of working days was used. The “double-selection” approach was performed also for the indirect costs, see above for more details. 

### Attributable cost of excess weight status.

The final regression model allowed to estimate the adjusted attributable costs and associated uncertainties of overweight and obesity compared to normal weight. Incremental costs were estimated at the individual level using the method of recycled predictions (also known as direct standardisation or g-computation) that allows to estimate the marginal effect from overweight and obesity on health care costs [16, 17]. The coefficients of the regression model were used to 1) predict health care costs for each respondent using the BMI from their reported weight and height; 2) predict health care cost assuming all respondents did have a normal BMI, keeping all other characteristics as observed; 3) calculate the individual incremental cost of obesity as the difference of an individual’s predicted costs assuming they were affected by obesity or overweight and assuming they had normal weight; and 4) calculate the attributable cost of obesity as the population survey-weighted average of the individual incremental cost. In order to jointly reflect prediction and survey uncertainty, means and CIs by BMI-classes were computed via bootstrapping with 1000 replicates and 1000 Monte Carlo simulations drawn per replication (for the survey design), leading to 1000*1000 interactions. In addition, total direct costs were calculated multiplying the average incremental cost by the proportion of individuals with overweight and obesity in the total adult population on the 1^st^ of January 2018 (*N* = 9,074,575) [18]. According to the BHIS2018, 33.4% and 15.9% of the Belgian adult population were respectively affected by overweight and obesity. Total indirect costs were calculated multiplying the average incremental absenteeism cost by the proportion of individuals with overweight and obesity in the total population with a paid job according to BHIS2013 (*N *= 3,906,170) [18], namely 33.9% and 11.3% of the Belgian population with a paid job were respectively affected by overweight and obesity.

### Relative contribution of chronic conditions

To investigate the relative importance of chronic conditions contributing to differences in health expenditure of persons with obesity and overweight compared to normal weight individuals, we evaluated how much of the attributable cost of excess weight can be attributed to each of 23 diseases. For this, we 1) extended the regression model for health care costs to also include the considered disease along with the covariates significant in a model with the disease as dependent variable; 2) used the model to predict health care cost assuming all respondents had a normal BMI, keeping all other characteristics (including disease status) as observed; 3) subtracted the predictions obtained in step 2 of the previous section from the obtained predictions; and 4) calculated how much of the attributable cost of obesity is due to the considered disease as the population survey-weighted average of the individual incremental cost obtained in the previous step, and dividing this by the average incremental cost of excess weight. For these analyses, the underweight population was omitted, considering that diseases related to underweight are commonly different from those related to excess weight. This method allowed to rank the diseases by their relative contribution to the incremental cost of obesity.

## Results

Table 1 describes socio-demographic characteristics and number of chronic conditions of the study population by BMI category. In 2013, 13.9% of the adult population aged 18 years and older was affected by obesity while 34.7% was affected by overweight. The individuals with overweight (excluding the population with obesity) comprised significantly more men than women and older than younger adults compared to the normal weight individuals. In the overweight and obesity BMI category, there were significantly more participants with lower educational levels and lower income, compared to the normal weight BMI category. The results show that a higher BMI is significantly associated with the number of chronic conditions. The prevalence of respondents with 3 or more chronic conditions increased from 2% in individuals with normal weight to 4% and 10% in participants respectively with overweight and obesity.

**Table 1:** Socio-demographic characteristics by body mass index category, Belgian population ≥ 18 years, BHIS 2013

	**Total**				**Underweight**				**Normal weight**				**Overweight**		**Obese**	
	N^(1)^		%^(2)^		N		%		N		%		N	%	N	%
**Total**	7,633		100		222		2.8		3,692		48.6		2,624	34.7	1,095	13.9
**Gender**																
Men	3,613		48.3		51		25.0		1,540		43.0		1,510	58.0	512	46.9
Women	4,020		51.7		171		75.0*		2,152		57.0		1,114	42.0*	583	53.1
**Age (in years)**																
18–34	1,978		27		113		64.2*		1,263		33.9		446	18.1*	156	15.6*
35–65	3,945		52		74		25.1*		1,781		49.8		1,461	55.7*	629	53.3*
≥ 66 years	1,710		21		35		10.7*		648		16.3		717	26.2*	310	28.1*
**Household education**																
No diploma/primary	779		9.3		19		4.4		253		5.6		311	11.0*	196	19.0*
Lower secondary	1,075		13.1		33		13.7		447		11.1		393	14.0*	202	18.0*
Higher secondary	2,486		34.7		70		37.8		1,159		34.5		870	34.0	387	36.6*
Higher education	3,191		42.9		99		44.1		1,780		48.9		1,014	41.0	298	26.4
**Household income**																
Quintile 1	1,398		16.9		55		24.5		589		14.8		489	16.9	265	22.5*
Quintile 2	1,136		16.1		30		13.5		505		14.2		396	16.2	205	22.6*
Quintile 3	1,347		20.9		35		20.4		652		22.0		466	20.5	194	18.1
Quintile 4	1,357		22.2		36		22.3		688		22.8		473	22.3	160	19.8
Quintile 5	1,496		23.9		41		19.3		792		26.1		500	24.1	163	17.0
**Number of chronic conditions**																
None	4,836		64		163		79		2,716		75		1,507	58	450	42
1	1,738		22		36		14		669		18		697	26*	336	30*
2	740		9		19		5		232		6		293	11*	196	17*
3 or more	315		4		4		1		71		2		127	4*	113	10*

^(1)^*N* = total number of participants in the survey

^(2)^Survey weighted prevalence

^*^ Prevalence significantly different *(p* < 0.001) relative to normal BMI-class

### Health care cost per BMI category

Table 2 shows the average yearly health care costs per capita from 2013–2017. The average yearly total health care cost increased from €2,246 per capita in normal weight individuals to €3,475 and €4,288 per capita in individuals respectively with overweight and obesity. In individuals with underweight the average health care expenditure was €3,387 per capita per year. Ambulatory care was 58% of total health care cost while 30% of the costs were for hospital care and 9.5% for reimbursed medicines obtained through pharmacies.

**Table 2:** Mean annual health expenses (in euro) per capita in function of body mass index categories, Belgian population ≥ 18 years, BHIS 2013 –IMA 2013–2017 (*N* = 7,633)

Health expenses	Total		Underweight BMI < 18		Normal weight 18 ≤ BMI < 24.5		Overweight 25 ≤ BMI < 29.5		Obese BMI ≥ 30	
	Mean	% of total	Mean	95% CI	Mean	95% CI	Mean	95% CI	Mean	95% CI
**Ambulatory care**	1,766	58	1,585	1,203–1,968	1,413	1,288–1,538	2,061	1,814–2,307	2,299	2,054–2,545
**Hospital care**	932	30	1,184	616–1,707	741	616–865	1,004	870–1,128	1,370	1,115–1,625
**Reimbursed medicines**	291	9.5	181	124–239	209	178–239	327	229–366	511	440–583
**Not specified**	77	2.5	87	56–118	63	52–74	83	73–94	107	88–126
**Total health care cost**	3,066	100	3,038	2,252–3,824	2,426	2,192–2,660	3,475	3,133–3,818	4,288	3,803–4,772

*CI* Confidence interval, *BHIS* Belgian Health Interview Survey, *IMA* Intermutualistic Agency

The unadjusted negative binomial regression model allowed to estimate the incremental costs of individuals with overweight and obesity versus normal weight individuals. The average yearly health care cost of overweight and obesity were significantly higher (i.e. 43% and 77% higher – *p* < 0.001) compared to normal weight (Table 3). Based on the method of recycled predictions, the mean incremental cost of overweight and obesity were €1,046 [CI: 677–1,445] and €1,870 [CI: 1,388–2,407] per capita, respectively, leading to a national incremental cost of €3,170,527,646 for the population with overweight and €2,698,190,204 for the population affected by obesity.

**Table 3:** Unadjusted health care costs in function of body mass index classes, Belgian population ≥ 18 years, BHIS 2013 – IMA2013-2017 (*N* = 7,633)

BMI categories	RR^(1)^	Standard error	*P*-value	95% CI	Mean incremental cost^(2)^
Underweight	1.25	0.17	0.111	0.91–1.65	627
Normal weight (reference)	1	—	—	—	—
Overweight	1.43	0.10	< 0.001	1.25–1.64	1,046
Obese	1.77	0.13	< 0.001	1.54–2.03	1,870
Constant	2,426	119	< 0.001	2,203–2,672	

^(1)^RR = expected value of the coefficient with normal weight as reference category

^(2)^Based on the method of recycled predictions

Since increased health expenses in individuals with overweight and obesity compared to individuals with normal weight are also related to socio-demographic factors and chronic health conditions, results presented in Table 3 needed to be adjusted. Univariate regression analysis of health care cost in function of BMI and each of the candidate predictors revealed that age, gender, household educational level and lack of physical activity likely had a confounding effect on BMI-related health care costs (*p* < 0.01). In addition, the multinomial regression of BMI categories as function of the candidate covariates revealed that the same predictors were significant (see Appendix Table 6). Moreover, considering that the average incremental cost of underweight was not statistically different from the normal weight one, the underweight population (*N* = 222) was excluded from further analysis.

The final multivariable model included age, gender, educational level and lack of physical activity as independent variables (Table 4). The high non-response rate on the physical activity questions in the BHIS resulted in a reduced sample size (*N* = 4,624; 60.6%). Since lack of physical activity is an important behavioural risk factor for chronic diseases, this indicator was kept as possible confounder in the multivariable model even if its inclusion would lead to a reduced sample size. In addition, sociodemographic characteristics of the reduced sample did not differ much from the original sample (see Appendix Table 7). Based on this model, the adjusted mean incremental cost for overweight and obesity was €651 [95% CI: €144-€1,084] and €1,015 [95%CI: €343–€1,697] per capita respectively. At national level, the adjusted incremental health care cost for the population affected by overweight was €1,864,464,355 and €1,464,742,302 for the population affected by obesity leading to a total incremental cost for Belgium of €3,329,206,657 related to excess body weight.

**Table 4:** Health care costs in function of body mass index categories adjusted for age, gender, household educational level and lack of physical activity, Belgian population ≥ 18 years – underweight population was excluded, BHIS 2013—IMA2013-2017 (*N* = 4,504)

	**Cost ratio**	**Standard error**	**P-value**	**95% CI**
**BMI categories**				
Normal weight (reference)	1	—	—	—
Overweight	1.24	0.11	0.010	1.05–1.47
Obese	1.36	0.14	0.003	1.11–1.65
**Age groups**				
18–34 years (reference)	1	—	—	—
35–64 years	1.44	0.20	0.009	1.09–1.89
≥ 65 years	2.62	0.41	< 0.001	1.93–3.55
**Gender**				
Male (reference)	1	—	—	—
Female	1.02	0.07	0.807	0.88–1.17
**Household educational level**				
No diploma or primary education	1.62	0.25	0.002	1.20–2.18
Lower secondary education	1.48	0.16	< 0.001	1.21–1.82
Higher secondary education	1.13	0.09	0.125	0.97–1.33
Higher education (reference)	1	—	—	—
**At risk due to lack of physical activity**				
Yes	1.37	0.12	< 0.001	1.15–1.62
No (reference)	1	—	—	—
Constant	1,164	152	< 0.001	901–1,503

### Disease associated health care cost related to overweight and obesity

**Fig. 2:**
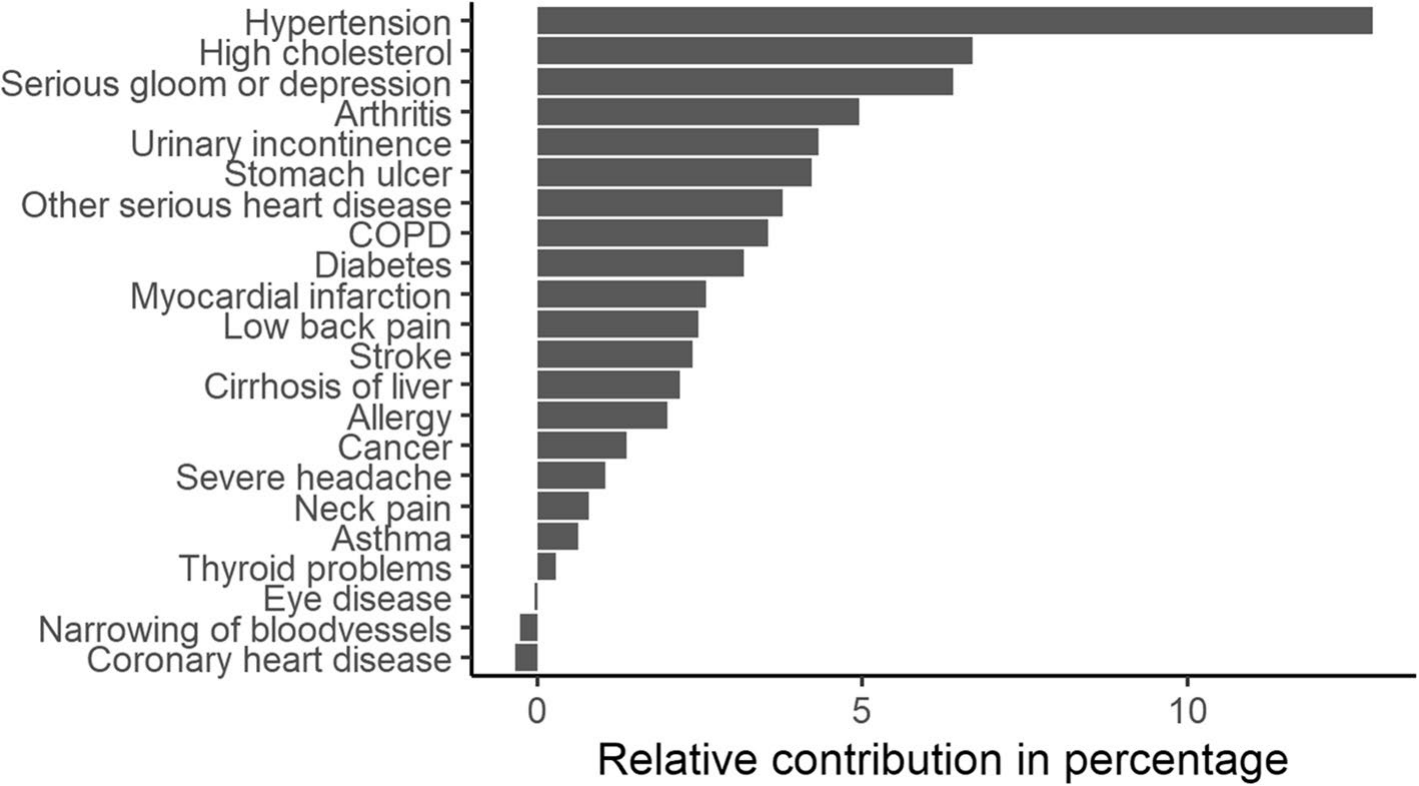
Relative contribution of chronic conditions to incremental costs of overweight and obesity, Belgian population ≥ 18 years, BHIS 2013-IMA2013-2017

Figure 2 presents the relative contribution of chronic conditions to the incremental direct costs of overweight and obesity among adults in Belgium. Appendix Table 8 shows the details of the fitted models of each chronic condition based on the significant confounders.

Twenty out of twenty-three chronic conditions included in the regression model had a positive contribution to the incremental cost of overweight and obesity (eye disease, coronary heart disease and narrowing of blood vessels showed a negative attributable cost) – see Appendix Table 10. Costs attributable to hypertension were by far the highest among all considered chronic conditions. The second highest relative cost was attributed to high cholesterol followed by serious gloom and depression.

### Cost of absenteeism

3,857 individuals were identified as the adult working population and included within the analysis (50.5% of the sample included in the previous analysis). The mean incremental cost of absenteeism in individuals with underweight and overweight was €360 and €242 per capita respectively but did not differ significantly from zero (Table 5). However, the cost increases significantly to €2,015 [95%CI: €179–€4,336] per capita in individuals with obesity. Based on the method of recycled predictions, productivity loss poses an incremental cost to the society of €320,082,750 and of €889,469,387 within the population with overweight and obesity respectively. The results for the adjusted model can be found in Appendix Table 9.

**Table 5:** Unadjusted costs of absenteeism in function of BMI-classes, Belgian working population ≥ 18 years, BHIS 2013 (*N* = 3,857)

BMI-classes	Cost ratio^(1)^	Std. error	P-value	95% CI
Underweight	1.19	0.48	0.671	(0.54–2.63)
Normal weight (reference)	1	—	—	—
Overweight	1.11	0.16	0.472	(0.83—1.48)
Obese	1.87	0.48	0.015	(1.13—3.09)
Constant	2,359	228	< 0.001	(1,952 – 2,851)

expected value of the coefficient with normal weight as reference category; *CI* Confidence intervals

The contribution of chronic conditions to the attributable absenteeism cost of excess weight was computed with the same methodology applied for the direct health care costs. 13 chronic diseases had a positive contribution to the incremental cost of absenteeism (Appendix Table 10). Arthritis, including rheumatoid arthritis and osteoarthritis, was the most important driver of the incremental cost of absenteeism in individuals with overweight and obesity, followed by hypertension and low back pain.

## Discussion

This study aimed to evaluate the societal impact of excess weight by estimating the direct and indirect costs associated with overweight and obesity among adults in Belgium. Our findings are based on a linkage of national health survey and health reimbursed cost data in Belgium for the years 2013 to 2017. Average yearly health care costs attributed to overweight and obesity were significantly higher (i.e. 43% and 77% higher) than average costs among individuals with normal weight. When adjusting for age, gender, household educational level and the lack of physical activity, the cost gap was reduced to 24% and 36% for respectively the population with overweight and obesity. Regarding the costs of absenteeism, individuals with obesity had a significantly higher cost compared to people with a normal weight (87% higher). Our results showed that in Belgium approximately €3.3 billion is spent yearly on average for direct healthcare costs due to excess body weight. It represents approximately 13.5% of the total yearly healthcare costs in Belgium and 10% of the yearly budget reserved to healthcare [19]. Yearly productivity loss due to work absenteeism poses an average cost of €1.2 billion that could be attributed to overweight and obesity in the Belgian working population.

In line with our estimates, OECD showed that the average healthcare expenditure for a person affected by obesity is 25% higher than for someone of normal weight [20]. Moreover, it is estimated that €70 billion are spent annually in Europe for healthcare and productivity loss due to obesity [21]. Other countries performed analysis similar to ours. Veiga (2008) compared two waves of the Portuguese National Health Survey (1996 vs 1999). Between the two waves, the total health care expenditures almost tripled for people with overweight (€133 vs €366 million) and more than doubled for people with obesity (€124 vs €261 million) [22]. Emery et al. (2007) estimated direct healthcare costs of obesity in France to be between €2.1 and €6.2 billion based on the Survey on Health and Social Protection of 2002 [23].

Considering that high BMI is associated with increased comorbidity, contributing to an increase in costs, we also investigated the relative contribution of different chronic diseases to the cost attributable to excessive body weight. In our study, hypertension constitutes by far the major contributor to incremental costs due to excess weight, followed by high cholesterol and serious gloom or depression. Different type of arthritis formed the main comorbidity driving the costs related to absenteeism, followed by hypertension and low back pain.

In a study conducted in the US looking at electronic medical records and claims, hypertensive diseases, dyslipidaemia, and osteoarthritis were the three most expensive obesity-related comorbidities at the population level; each responsible for $18 million annually. Moreover, it was found that hypertension and osteoarthritis were much more costly among individuals with obesity than those without obesity [24]. In Padula et al. (2014), total net expenditures of obesity and its comorbidities were calculated based on US claims in 2012. The combination of obesity and hypertension was the most common condition (inpatient and outpatient claims) accounting for a mean total cost of around $4,000, followed by obesity and diabetes and obesity and depression [25].

Our study provides valuable information on the extent of the societal impact that excessive weight status has in Belgium. The approach of recycled predictions has allowed us to compare direct and indirect healthcare costs among different BMI categories while adjusting for confounding by including important sociodemographic and health status covariates in the models. Our findings are also important from a health policy perspective, in the planning of strategies for health care cost containment. From a public health perspective, a sustainable approach towards effective prevention of the most impactful diseases is a more affordable strategy [26]. Public health programs to promote weight reduction and weight management among people affected by obesity and overweight play an important role in curbing the economic burden of different diseases. According to the state of health report of the EU countries, there are many modifiable behavioural risk factors related to overweight and obesity that could be improved. In Belgium, about 25% of people do not eat any vegetables and 45% any fruit daily. Moreover, Belgian adults are less physically active than those in many EU countries [27] and on average one third of their consumption is from ultra-processed food products [28].

We acknowledge some limitations within our study. First, there are some limitations that are intrinsic of the nature of our data sources. Self-reported data, deriving from national surveys, is subject to recalling and social desirability biases. This might have influenced primarily the reporting of height and weight, known to be a source of underestimation within the BHIS [29], as well as the amount of non-responses for heavy daily smoking and lack of physical activity that led to a considerable reduction of the sample size. In addition, participants with a low socio-economic status are more likely to leave questions without answering [30] and to be subject to excess weight status [31]. This might have led to underestimation of the prevalence of overweight and obesity. The number of days absent from work estimation were also gathered from self-reported, subjecting our data to uncertainty due to recall bias. Nevertheless, surveys represent an essential source of information for lifestyle characteristics, like smoking, eating habits, and chronic diseases that remain frequently un-diagnosed so they are difficult to grasp with other types of data sources (e.g. low back pain). With regard to cost data, national claims data collected at population-level do not include services that are not covered by the insurance (e.g. ambulant psychotherapy, limited reimbursements for physiotherapy). Even so, administrative data are an essential source for investigating the financial burden of healthcare. We chose to compute the cost of absenteeism using the human capital approach, being the most suitable method for our data, but this might have overestimated the costs. Other methods, such as friction cost approach, could be explored to limit this overestimation in further analysis. The inclusion of the costs of the previous year in the analysis was considered to not be pertinent to compute incremental costs for overweight and obesity. These choice is more comment in case of cute onset of disease/injury [32, 33]. A further limitation is the possibility of residual confounding bias in the cost estimation. We tried to overcome this by increasing the chance of detecting measured confounders via the double-selection process, but it may well be that certain important confounders were lacking from the database. The analysis of the relative contribution of diseases is especially vulnerable to this, as it additionally needs adjustment for common causes of disease and health care costs, and ignores that the considered diseases may mutually influence each other. In addition, some variables suffered from a high rate of non-responses, decreasing the sample size and possibly introducing bias. Nevertheless, comparing the socio-demographic characteristics of the initial sample and those of the reduced one showed no particular difference (Appendix Table 6). In future analyses, multiple imputation could be used for addressing potential selection bias and for lessening the information loss that results from the reduced sample size. Authors are also aware that in the observed 5-years after filling in the survey might have lost weight and change BMI status. Nevertheless, we were interested in looking in the long term chronic effects of excess weight, that is why we were interested in having a follow-up as long as possible. This limitation highlights the need and importance of cohort studies that allow to follow-up participants through time.

Considering that there is currently no national nutrition and physical activity health plan in Belgium [34], our estimates can inform policy makers and ease evidence-based interventions. In 2019, the WaIST project was initiated in Belgium aiming to provide proactive policy support for the prevention of excessive weight gain [35]. As part of this project health impact assessment will be used to model different internationally recommended health policies tackling overweight and obesity. Acting on the risk factors will help to reduce a cumbersome burden carried by our society largely affected by non-communicable diseases.

## Conclusions

Based on national health and financial estimates, we found that high BMI has a substantial societal economic burden in Belgium. We estimated that every year at least €4.5 billion are spent to cover the direct and indirect costs related to overweight and obesity. Policies and interventions are urgently needed to reduce the prevalence of overweight and obesity thereby decreasing these substantial costs.

## Supplementary Information

Below is the link to the electronic supplementary material.

**Additional file 1: Table 6.** Multinomial regression of body mass index classes (normal weight category as reference) in function of age, gender, educational level and lack of physical activity, Belgian population ≥ 18 years – underweight population was excluded, BHIS2013-IMA2013-2017 – the model includes the confounders that were significant after backwards stepwise elimination (N= 4,504). **Table 7.** Socio-demographic characteristics by body mass index category, Belgian population ≥18 years, health interview survey 2013 for population included in the multivariate regression (with no missing values in physical activity and educational level).** Table 8.** Chronic disease in function of available confounders – coefficient (standard error), Belgian population ≥ 18 years – underweight population was excluded, BHIS 2013-IMA2013-2017 – the model includes the confounders that were significant after backwards stepwise elimination.** Table 9.** Cost of absenteeism in function of body mass index classes adjusted for age, gender, educational level, nationality, lack of physical activity, tobacco use and daily intake of sugared drinks - Belgian population ≥ 18 years, BHIS 2013-IMA2013-2017 (N= 2,480).** Table 10.** Relative contribution of chronic disease to the direct and indirect costs (in percentage).

## Data Availability

All data generated during this study is included in the appendix of this article.

## Abbreviations

BHES: Belgian health examination survey
BHIS: Belgian health interview survey
BMI: Body mass index
CI: Confidence interval
DALY: Disability-adjusted life years
IMA: Intermutualistic agency
RR: Risk ratio
