# Supplementary material for: Health care costs and lost productivity costs related to excess weight in Belgium
Source: BMC Public Health. 2022 Sep 6;22:1693. doi: 10.1186/s12889-022-14105-9 (PMC9450378; doi:10.1186/s12889-022-14105-9)
Supplement: Supplementary file 1 — Additional file 1: Table 6. Multinomial regression of body mass index classes (normal weight category as reference) in function of age, gender, educational level and lack of physical activity, Belgian population ≥ 18 years – underweight population was excluded, BHIS2013-IMA2013-2017 – the model includes the confounders that were significant after backwards stepwise elimination (N= 4,504). Table 7. Socio-demographic characteristics by body mass index category, Belgian population ≥18 years, health interview survey 2013 for population included in the multivariate regression (with no missing values in physical activity and educational level). Table 8. Chronic disease in function of available confounders – coefficient (standard error), Belgian population ≥ 18 years – underweight population was excluded, BHIS 2013-IMA2013-2017 – the model includes the confounders that were significant after backwards stepwise elimination. Table 9. Cost of absenteeism in function of body mass index classes adjusted for age, gender, educational level, nationality, lack of physical activity, tobacco use and daily intake of sugared drinks - Belgian population ≥ 18 years, BHIS 2013-IMA2013-2017 (N= 2,480). Table 10. Relative contribution of chronic disease to the direct and indirect costs (in percentage). [file 12889_2022_14105_MOESM1_ESM.docx]

SUPPLEMENTARY FILE

Table 6: Multinomial regression of body mass index classes (normal weight category as reference) in function of age, gender, educational level and lack of physical activity, Belgian population ≥ 18 years – underweight population was excluded, BHIS2013-IMA2013-2017 – the model includes the confounders that were significant after backwards stepwise elimination (N= 4,504)

|  | Overweight | | | Obesity | | |
| --- | --- | --- | --- | --- | --- | --- |
|  | Coefficient | Std. error | P-value | Coefficient | Std. error | P-value |
| **Age-groups** |  |  |  |  |  |  |
| 18-34 years (base) | |  |  |  |  |  |
| 35-64 years | 0.56 | 0.12 | <0.001 | 0.86 | 0.19 |  |
| ≥ 65 years | 0.85 | 0.14 | <0.001 | 0.69 | 0.23 |  |
| **Gender** |  |  |  |  |  |  |
| Male (base) |  |  |  |  |  |  |
| Female | -0.74 | 0.08 | <0.001 | -0.22 | 0.14 | >0.100 |
| **Educational level** |  |  |  |  |  |  |
| No diploma or primary education (base) | |  |  |  |  |  |
| Lower secondary education | 0.50 | 0.23 | 0.030 | -0.75 | 0.26 | 0.006 |
| Higher secondary education | -0.72 | 0.20 | <0.001 | -1.20 | 0.25 | <0.001 |
| Higher education | -0.85 | 0.19 | <0.001 | -1.68 | 0.29 |  |
| **At risk due to lack of physical activity** | | |  |  |  |  |
| Yes | 0.34 | 0.10 | 0.001 | 0.80 | 0.16 | <0.001 |
| No (base) |  |  |  |  |  |  |
| Constant | 0.19 | 0.22 | >0.100 | -0.84 | 0.26 | 0.002 |

Table 7: Socio-demographic characteristics by body mass index category, Belgian population ≥18 years, health interview survey 2013 for population included in the multivariate regression (with no missing values in physical activity and educational level)

|  | Total | | | | Underweight | | | Normal weight | | Overweight | | Obese | |
| --- | --- | --- | --- | --- | --- | --- | --- | --- | --- | --- | --- | --- | --- |
|  | N^(1)^ | | %^(2)^ | | N | | % | N | % | N | % | N | % |
| **Total** | 4,624 | | 100 | | 120 | | 2.5 | 2,262 | 49.1 | 1,600 | 35.5 | 642 | 12.9 |
| **Gender** |  | |  | |  | |  |  |  |  |  |  |  |
| Men | 2,178 | | 48.1 | | 21 | | 17.9 | 923 | 42.4 | 942 | 58.7 | 292 | 44.8 |
| Women | 2,446 | | 51.9 | | 99 | | 82.1 | 1,339 | 57.7 | 658 | 44.8 | 350 | 55.2 |
| **Age** |  | |  | |  | |  |  |  |  |  |  |  |
| 18-34 years | 1,135 | | 25.3 | | 61 | | 62.7 | 715 | 30.5 | 272 | 18.4 | 87 | 14.4 |
| 35-65 years | 2,458 | | 54.4 | | 41 | | 26.3 | 1,140 | 53.0 | 892 | 55.9 | 385 | 61.1 |
| ≥ 66 years | 1,031 | | 20.3 | | 18 | | 11.0 | 407 | 16.5 | 436 | 25.8 | 170 | 24.5 |
| **Household education** | |  | |  | |  |  |  |  |  |  |  |  |
| No diploma/primary | 342 | | 7.1 | | 6 | | 1.9 | 100 | 3.8 | 151 | 9.4 | 85 | 15.4 |
| Lower secondary | 596 | | 12.3 | | 15 | | 15.1 | 242 | 9.8 | 221 | 13.2 | 118 | 17.8 |
| Higher secondary | 1,454 | | 33.1 | | 36 | | 37.7 | 683 | 33.0 | 508 | 32.6 | 227 | 34.4 |
| Higher education | 2,232 | | 47.5 | | 63 | | 45.3 | 1,237 | 53.4 | 720 | 44.9 | 212 | 32.5 |
| **Household income** | |  | |  | |  |  |  |  |  |  |  |  |
| Quintile 1 | 607 | | 12.3 | | 23 | |  | 258 |  | 204 |  | 122 |  |
| Quintile 2 | 630 | | 14.3 | | 17 | |  | 277 |  | 233 |  | 103 |  |
| Quintile 3 | 875 | | 20.9 | | 21 | |  | 419 |  | 302 |  | 133 |  |
| Quintile 4 | 979 | | 24.7 | | 28 | |  | 508 |  | 331 |  | 112 |  |
| Quintile 5 | 1,127 | | 27.8 | | 7 | |  | 199 |  | 140 |  | 60 |  |
| **Number of chronic conditions** |  | |  | |  | |  |  |  |  |  |  |  |
| None | 2,887 | | 63.9 | | 88 | | 76.4 | 1,634 |  | 899 | 57.4 | 266 | 41.6 |
| 1 | 1,121 | | 23.5 | | 17 | | 16.1 | 453 |  | 457 | 26.9 | 194 | 31.2 |
| 2 | 455 | | 9.4 | | 12 | | 6.7 | 137 |  | 184 | 12.1 | 122 | 19.4 |
| 3 or more | 160 | | 3.1 | | 3 | | 0.7 | 37 |  | 60 | 3.5 | 60 | 7.8 |

Table 8: Chronic disease in function of available confounders – coefficient (standard error), Belgian population ≥ 18 years – underweight population was excluded, BHIS 2013-IMA2013-2017 – the model includes the confounders that were significant after backwards stepwise elimination

|  | Allergy | Asthma | Cancer | Cirrhosis | Coronary heart disease | Chronic bronchitis and COPD | Diabetes | Eye disease | High cholesterol | Hyper-tension | Lower Back Pain | Myocardial infarction |
| --- | --- | --- | --- | --- | --- | --- | --- | --- | --- | --- | --- | --- |
| **Age-groups** |  |  |  |  |  |  |  |  |  |  |  |  |
| 18-34 years (base) |  |  |  |  |  |  |  |  |  |  |  |  |
| 35-64 years |  |  | 2.85 (0.61) | 16.7 (0.27) | 1.80 (1.04) | 1.05 (0.53) | 1.41 (0.43) | 1.34 (0.35) | 1.72 (0.26) | 2.46 (0.29) | 1.88 (0.36) | 15.52 (0.40) |
| ≥ 65 years |  | -0.57 (0.30) | 4.13 (0.62) | 16.5 (0.46) | 3.06 (1.05) | 1.58 (0.50) | 2.18 (0.43) | 3.35 (0.32) | 2.77 (0.27) | 3.25 (0.30) | 2.81 (0.34) | 17.88 (0.37) |
| **Gender** |  |  |  |  |  |  |  |  |  |  |  |  |
| Male (base) |  |  |  |  |  |  |  |  |  |  |  |  |
| Female | 0.32 (010) |  | 0.71 (0.26) |  |  |  | -0.37 (0.19) |  |  |  | 0.63 (0.11) |  |
| **Educational level** |  |  |  |  |  |  |  |  |  |  |  |  |
| No diploma or primary education (base) |  |  |  |  |  |  |  |  |  |  |  |  |
| Lower secondary education |  |  |  |  |  |  |  |  |  |  | -0.37 (0.21) |  |
| Higher secondary education |  |  |  |  |  |  | -1.05 (0.27) |  |  | -0.41 (0.22) | -0.47 (0.19) |  |
| Higher education |  |  |  |  |  |  | -1.14 (0.26) | -0.53 (0.23) |  | -0.81 (0.23) | -0.92 (0.19) |  |
| **Income** |  |  |  |  |  |  |  |  |  |  |  |  |
| Quantile 1 (base) |  |  |  |  |  |  |  |  |  |  |  |  |
| Quantile 2 |  |  |  |  |  |  |  |  |  |  |  | -1.37 (0.78) |
| Quantile 3 |  |  |  |  |  | -0.71 (0.33) |  |  |  |  |  | -2.48 (0.75) |
| Quantile 4 | 0.51 (0.18) |  |  |  |  | -0.61 (0.36) |  |  |  |  |  |  |
| Quantile 5 | 0.45 (0.18) |  |  |  |  | -1.02 (0.37) |  |  |  |  |  | -2.34 (0.72) |
| **Nationality** |  |  |  |  |  |  |  |  |  |  |  |  |
| Belgian (base) |  |  |  |  |  |  |  |  |  |  |  |  |
| Non-Belgian |  |  |  |  |  |  |  |  |  |  |  | -16.45 (0.65) |
| Non-EU |  |  |  |  | -13.54 (0.31) |  |  |  | -11 (0.48) |  |  | 0.98 (0.56) |
| **At risk due to lack of physical activity** |  |  |  |  |  |  |  |  |  |  |  |  |
| Yes |  | 0.93 (0.20) |  | 0.73 (0.73) | 1.25 (0.40) | 0.87 (0.25) | 0.48 (0.20) |  | 0.34 (0.12) | 0.35 (0.16) | 0.24 (0.12) |  |
| No (base) |  |  |  |  |  |  |  |  |  |  |  |  |
| **Daily intake of sugared drinks** |  |  |  |  |  |  |  |  |  |  |  |  |
| Yes | 0.28 (0.13) |  |  | -2.54 (1.04) |  |  | -0.60 (0.28) |  |  | -0.38 (0.17) |  |  |
| No (base) |  |  |  |  |  |  |  |  |  |  |  |  |
| **Over-consumption of alcohol** |  |  |  |  |  |  |  |  |  |  |  |  |
| Yes |  |  |  |  |  |  |  |  | 0.50 (0.21) | 0.74 (0.20) |  |  |
| No (base) |  |  |  |  |  |  |  |  |  |  |  |  |
| **Heavy daily smoking** |  |  |  |  |  |  |  |  |  |  |  |  |
| Yes |  |  |  | 1.01 (0.59) |  | 1.26 (0.37) |  |  |  |  |  |  |
| No (base) |  |  |  |  |  |  |  |  |  |  |  |  |
| Constant | -2.26 (0.17) |  | -7.58 (0.61) | -21.51 (0.17) | -7.11 (0.99) |  | -3.65 (0.48) | -4.73 (0.35) | -3.46 (0.25) | -3.94 (0.39) | -3.36 (0.37) |  |

**Table 8 (continued)**

|  | Narrowing blood-vessels | Neck pain | Other serious heart disease | Serious gloom | Severe headache | Stomach ulcer | Stroke | Arthritis | Thyroid | Urinary incontinence |
| --- | --- | --- | --- | --- | --- | --- | --- | --- | --- | --- |
| **Age-groups** |  |  |  |  |  |  |  |  |  |  |
| 18-34 years (base) |  |  |  |  |  |  |  |  |  |  |
| 35-64 years | 1.98 (1.03) | 1.51 (0.17) | 2.07 (0.67) | 1.13 (0.24) |  | 1.28 (0.37) | 17.04 (0.29) | 1.71 (0.21) | 0.90 (0.19) | 3.11 (0.94) |
| ≥ 65 years | 3.06 (1.03) | 1.89 (0.18) | 3.19 (0.67) | 0.60 (0.32) |  | 1.69 (0.40) | 17.50 (0.34) | 2.80 (0.21) | 1.27 (0.20) | 3.98 (0.94) |
| **Gender** |  |  |  |  |  |  |  |  |  |  |
| Male (base) |  |  |  |  |  |  |  |  |  |  |
| Female |  | 0.55 (0.10) | -0.57 (0.32) | 0.47 (0.18) | 1.18 (0.13) |  |  | 0.60 (0.08) | 1.79 (0.16) | 0.58 (0.24) |
| **Educational level** |  |  |  |  |  |  |  |  |  |  |
| No diploma or primary education (base) |  |  |  |  |  |  |  |  |  |  |
| Lower secondary education |  |  |  |  |  |  |  | -0.33 (0.16) |  | -0.71 (0.37) |
| Higher secondary education |  |  |  |  |  |  |  | -0.33 (0.14) |  | -1.03 (0.37) |
| Higher education |  |  |  | -0.67 (0.31) |  |  |  | -0.75 (0.14) |  | -1.76 (0.40) |
| **Income** |  |  |  |  |  |  |  |  |  |  |
| Quantile 1 (base) |  |  |  |  |  |  |  |  |  |  |
| Quantile 2 |  |  |  |  |  |  |  |  |  |  |
| Quantile 3 |  |  |  |  |  |  |  |  |  |  |
| Quantile 4 |  |  |  |  |  | -0.64 (0.35) |  |  |  |  |
| Quantile 5 |  |  |  | -0.97 (0.35) |  | -1.21 (0.42) | -1.64 (0.80) |  |  |  |
| **Nationality** |  |  |  |  |  |  |  |  |  |  |
| Belgian (base) |  |  |  |  |  |  |  |  |  |  |
| Non-Belgian | -1.50 (0.81) |  | -1.21 (0.63) |  |  |  | -1.82 (1.05) |  |  | -2.23 (0.83) |
| Non-EU | 0.20 (0.64) |  | 0.29 (0.62) |  | 1.16 (0.35) |  | -17.03 (0.37) |  |  |  |
| **At risk due to lack of physical activity** |  |  |  |  |  |  |  |  |  |  |
| Yes | 0.87 (0.30) |  | 0.62 (0.31) | 0.40 (0.20) |  |  | 1.37 (0.42) |  |  | 1.10 (0.25) |
| No (base) |  |  |  |  |  |  |  |  |  |  |
| **Daily intake of sugared drinks** |  |  |  |  |  |  |  |  |  |  |
| Yes |  | 0.22 (0.11) |  |  | 0.42 (0.15) |  |  |  |  |  |
| No (base) |  |  |  |  |  |  |  |  |  |  |
| **Over-consumption of alcohol** |  |  |  |  |  |  |  |  |  |  |
| Yes |  |  | -1.10 (0.63 | 0.65 (0.33) |  |  |  |  |  | -1.21 (0.50) |
| No (base) |  |  |  |  |  |  |  |  |  |  |
| **Heavy daily smoking** |  |  |  |  |  |  |  |  |  |  |
| Yes |  |  |  | 0.54 (0.27) | 0.62 (0.22) | 0.57 (0.34) |  |  |  |  |
| No (base) |  |  |  |  |  |  |  |  |  |  |
| Constant | -6.53 (1.04) | -3.70 (0.17) | -6.16 (0.67) | -3.29 (0.38) | -3.23 (0.12) | -4.62 (0.41) | -20.90 (0.43) | -3.85 (0.26) | -4.77 (0.21) | -6.42 (1.00) |

Table 9: Cost of absenteeism in function of body mass index classes adjusted for age, gender, educational level, nationality, lack of physical activity, tobacco use and daily intake of sugared drinks - Belgian population ≥ 18 years, BHIS 2013-IMA2013-2017 (N= 2,480)

|  | Cost ratio | Std. error | P-value | | 95% CI | | Mean incremental cost | |
| --- | --- | --- | --- | --- | --- | --- | --- | --- |
| **BMI-classes** |  |  |  | |  | |  | |
| Underweight | 1.27 | 0.60 | >0.100 | | 0.50-3.22 | | 718 | |
| Normal weight (base) | 1 |  |  |  | |  | |  |
| Overweight | 1.30 | 0.21 | >0.100 | | 0.95–1.79 | | 650 | |
| Obese | 1.78 | 0.62 | 0.097 | | 0.90–1.52 | | 1,888 | |
| **Age-groups** |  |  |  | |  | |  | |
| 18-34 years (base) | 1 |  |  | |  | |  | |
| 35-64 years | 0.94 | 0.20 | >0.100 | | 0.62-1.43 | |  | |
| ≥ 65 years | 0.28 | 0.22 | >0.100 | | 0.06-1.30 | |  | |
| **Gender** |  |  |  | |  | |  | |
| Male (base) | 1 |  |  | |  | |  | |
| Female | 1.90 | 0.33 | <0.001 | | 1.35–2.66 | |  | |
| **Educational level** |  |  |  | |  | |  | |
| No diploma or primary education | 1.67 | 0.71 | >0.100 | | 0.72-3.83 | |  | |
| Lower secondary education | 1.01 | 0.29 | >0.100 | | 0.57-1.79 | |  | |
| Higher secondary education | 1.25 | 0.24 | >0.100 | | 0.86-1.81 | |  | |
| Higher education (base) | 1 |  |  | |  | |  | |
| **At risk due to lack of physical activity** | | | | |  | |  | |
| Yes | 1.19 | 0.26 | >0.100 | | 0.77-1.83 | |  | |
| No (base) | 1 |  |  | |  | |  | |
| **Nationality** |  |  |  | |  | |  | |
| Belgian (base) |  |  |  | |  | |  | |
| Non-Belgian | 0.58 | 0.18 | 0.081 | | 0.32-1.07 | |  | |
| Non-EU | 0.95 | 0.45 | >0.100 | | 0.37-2.41 | |  | |
| **Heavy daily smoking** |  |  |  | |  | |  | |
| Yes | 1.36 | 0.40 | >0.100 | | 0.76-2.43 | |  | |
| No (base) | 1 |  |  | |  | |  | |
| **Daily intake of sugared drinks** | |  |  | |  | |  | |
| Yes | 1.21 | 0.22 | >0.100 | | 0.85-1.73 | |  | |
| No (base) | 1 |  |  | |  | |  | |
| Constant | 1,414 | 295 | <0.001 | | 939-2,128 | |  | |

| Table 10: Relative contribution of chronic disease to the direct and indirect costs (in percentage)   \| **Disease** \| **Relative contribution to the direct cost** \| **Relative contribution to the cost of absenteeism** \| \| --- \| --- \| --- \| \| Hypertension \| 12.85 \| 5.50 \| \| High cholesterol \| 6.68 \| 2.94 \| \| Serious gloom or depression \| 6.39 \| 2.25 \| \| Arthritis \| 4.95 \| 10.00 \| \| Urinary incontinence \| 4.32 \| 0.26 \| \| Stomach ulcer \| 4.22 \| 0.09 \| \| Other serious heart disease \| 3.77 \| 2.54 \| \| COPD \| 3.55 \| 2.79 \| \| Diabetes \| 3.18 \| -0.31 \| \| Myocardial infarction \| 2.60 \| -0.60 \| \| Low back pain \| 2.48 \| 5.13 \| \| Stroke \| 2.39 \| 3.78 \| \| Cirrhosis of liver \| 2.20 \| 0.56 \| \| Allergy \| 1.99 \| -1.05 \| \| Cancer \| 1.37 \| -2.70 \| \| Severe headache \| 1.05 \| -2.01 \| \| Neck pain \| 0.80 \| -1.01 \| \| Asthma \| 0.63 \| -0.18 \| \| Thyroid problems \| 0.28 \| -0.26 \| \| Eye disease \| -0.05 \| 0.71 \| \| Narrowing of bloodvessels \| -0.26 \| -1.18 \| \| Coronary heart disease \| -0.35 \| -1.43 \| \|  \|  \| |
| --- | --- | --- | --- | --- | --- | --- | --- | --- | --- | --- | --- | --- | --- | --- | --- | --- | --- | --- | --- | --- | --- | --- | --- | --- | --- | --- | --- | --- | --- | --- | --- | --- | --- | --- | --- | --- | --- | --- | --- | --- | --- | --- | --- | --- | --- | --- | --- | --- | --- | --- | --- | --- | --- | --- | --- | --- | --- | --- | --- | --- | --- | --- | --- | --- | --- | --- | --- | --- | --- | --- | --- |
